# Rapid Antimicrobial Susceptibility Testing Using Direct Centrifugation Significantly Reduces Turnaround Time While Maintaining High Diagnostic Accuracy in Bloodstream Infections

**DOI:** 10.3390/diagnostics16183007

**Published:** 2026-09-17

**Authors:** Chi-Yen Wong, Swee-Siang Wong, Wen-Ling Yen, Li-Min Chang, Wei-Shang Hsih, Chien-Chun Yang, Ya-Yuan Mao, Ming-Hsueh Tsai, Ting-I Wu, Yu-Che Cheng

**Affiliations:** 1 Department of Clinical Pathology, Cathay General Hospital, Taipei 10630, Taiwan; ja74@cgh.org.tw (C.-Y.W.);; 2 Department of Infectious Diseases, Cathay General Hospital, Taipei 10630, Taiwan; 3 Proteomics Laboratory, Cathay Medical Research Institute, Cathay General Hospital, Taipei 10630, Taiwan; 4 Department of Biomedical Sciences and Engineering, National Central University, Jhongli 32001, Taiwan; 5 Department of Infection Control, Cathay General Hospital, Taipei 10630, Taiwan

**Keywords:** antimicrobial susceptibility testing, turnaround time, bloodstream infections

## Abstract

**Background/Objectives:** Bloodstream infection-associated sepsis and septic shock remain major causes of in-hospital mortality. Because conventional antimicrobial susceptibility testing (AST) requires 48–72 h, earlier susceptibility results may facilitate timely antimicrobial optimization. This study evaluated the performance and clinical utility of a direct centrifugation-based AST method compared with the standard workflow. **Methods:** A total of 328 monomicrobial positive blood culture isolates, comprising 168 Gram-negative bacteria and 160 Gram-positive cocci, were analyzed. Essential agreement (EA), categorical agreement (CA), very major errors (VMEs), major errors (MEs), minor errors (mEs), and turnaround time (TAT) were compared between the two methods. **Results:** For Gram-negative bacteria, the direct method achieved an EA of 97.77% and CA of 98.53%, with VME, ME, and mE rates of 0.81%, 0.14%, and 0.60%, respectively. Particularly high concordance was observed for Escherichia coli and Klebsiella pneumoniae. For Gram-positive cocci, the EA and CA were 93.29% and 95.91%, respectively, with VME, ME, and mE rates of 3.47%, 1.08%, and 0.94%, respectively. Although overall agreement remained high, the GPC VME rate slightly exceeded the predefined 3% criterion, and elevated species-specific VME rates were observed for several organisms. The direct centrifugation method significantly reduced the mean TAT from 48.74 to 25.13 h, corresponding to an approximately 23.61 h reduction. **Conclusions:** The direct centrifugation method substantially shortened AST reporting time while maintaining high overall agreement with the standard workflow, particularly for Gram-negative bacteria. Further validation is warranted for Gram-positive and organism–antimicrobial combinations associated with elevated VME rates before routine clinical implementation.

## 1. Introduction

Bloodstream infection (BSI) is a serious and potentially life-threatening condition associated with substantial morbidity, mortality, and healthcare burden worldwide [1]. Once pathogens invade the bloodstream, patients may rapidly progress to sepsis, septic shock, multiple organ dysfunction, and death, particularly in elderly individuals, immunocompromised patients, and those with severe comorbidities [2]. In addition to its high fatality, BSI is also associated with prolonged hospitalization, intensive care unit admission, and increased medical expenditure [3]. The growing emergence of multidrug-resistant organisms, particularly methicillin-resistant *Staphylococcus aureus* and resistant Gram-negative bacteria such as *Escherichia coli* and *Klebsiella pneumoniae*, has further increased the complexity of treatment and worsened clinical outcomes [4]. Although blood culture remains the reference standard for diagnosing BSI, its clinical utility is constrained by slow turnaround time, often delaying pathogen-directed treatment and contributing to inappropriate antimicrobial use [5].

Early and accurate diagnosis of BSI is crucial because delayed initiation of appropriate antimicrobial therapy is strongly associated with increased mortality [6]. However, conventional blood culture, which remains the current diagnostic gold standard, has several important limitations, including low sensitivity in patients with prior antibiotic exposure, long turnaround time, and susceptibility to contamination. Moreover, the low concentration of circulating pathogens in peripheral blood poses a major technical challenge for direct pathogen detection [7]. Importantly, bloodstream infection is closely related to, but not synonymous with, sepsis, since not all septic patients have detectable bacteremia and negative blood culture results do not exclude clinically significant infection [8]. The rising incidence and mortality of sepsis are driven by multiple factors, including population aging, increasing prevalence of chronic diseases, immunosuppression, and the expanding use of invasive medical interventions [9]. A retrospective analysis in the United States from 2009 to 2014 demonstrated a marked increase in the incidence of sepsis and septic shock [10]. Timely diagnosis and early initiation of appropriate antimicrobial therapy are critical determinants of survival in patients with sepsis. Previous studies have consistently demonstrated that delays in diagnosis and treatment are associated with increased mortality [11]. However, under current laboratory workflows, it typically takes around 48 h from a positive blood culture to complete organism identification and antimicrobial susceptibility testing turnaround time (TAT). Reducing turnaround time could substantially improve clinical outcomes by enabling earlier targeted therapy [12].

Currently, widely used automated blood culture systems, including the BacT/ALERT^®^ system (bioMérieux) [13], BD BACTEC system [14], and VersaTREK^®^ system [15], can detect microbial growth in blood samples within hours to days. Nevertheless, these systems do not provide immediate species identification or susceptibility results. Positive blood culture samples must undergo subculture for 16–24 h to obtain isolated colonies prior to downstream Mass spectrometry identification and antimicrobial susceptibility testing (AST). Therefore, strategies that shorten the diagnostic workflow after blood culture positivity are needed to accelerate antimicrobial susceptibility testing and support earlier optimization of antimicrobial therapy.

Direct antimicrobial susceptibility testing from positive blood cultures has been investigated for more than two decades as a means of eliminating the subculture step. Recently, direct disk diffusion has been standardized by the European Committee on Antimicrobial Susceptibility Testing as the rapid antimicrobial susceptibility testing (RAST) method, with organism- and antimicrobial-specific breakpoints for defined incubation periods [16]. The Clinical and Laboratory Standards Institute has also incorporated direct disk diffusion procedures and breakpoints for selected organism–antimicrobial combinations into the M100 document [17]. Thus, the general feasibility of direct AST from positive blood cultures is well established. Nevertheless, standardized direct disk diffusion is limited to specific organism–antimicrobial combinations and reading times. Evidence supporting centrifugation-based bacterial recovery for routine AST across both Gram-negative and Gram-positive bloodstream isolates also remains limited. Here, we investigated whether a simple centrifugation-based preparation method could achieve acceptable essential and categorical agreement, identify organism-specific limitations, and meaningfully reduce turnaround time under routine clinical laboratory conditions.

## 2. Materials and Methods

### 2.1. Study Population and Sample Collection

Positive blood culture (PBC) bottles showing a single microbial morphology by Gram staining were prospectively collected between 8 January and 30 September 2025. In total, 328 clinical isolates were enrolled in this study, including 168 Gram-negative and 160 Gram-positive bacterial isolates.

### 2.2. Ethical Approval

This study was reviewed and approved by the Institutional Review Board of Cathay General Hospital (CGH-IRB No. CGH-P113065). The date of approval was 8 January 2025. The requirement for written informed consent was waived by the IRB because the study used residual clinical specimens and involved no additional intervention or risk to the participants. The study was conducted in accordance with the Declaration of Helsinki and applicable institutional guidelines.

### 2.3. Direct Centrifugation Method

**Figure 1:**
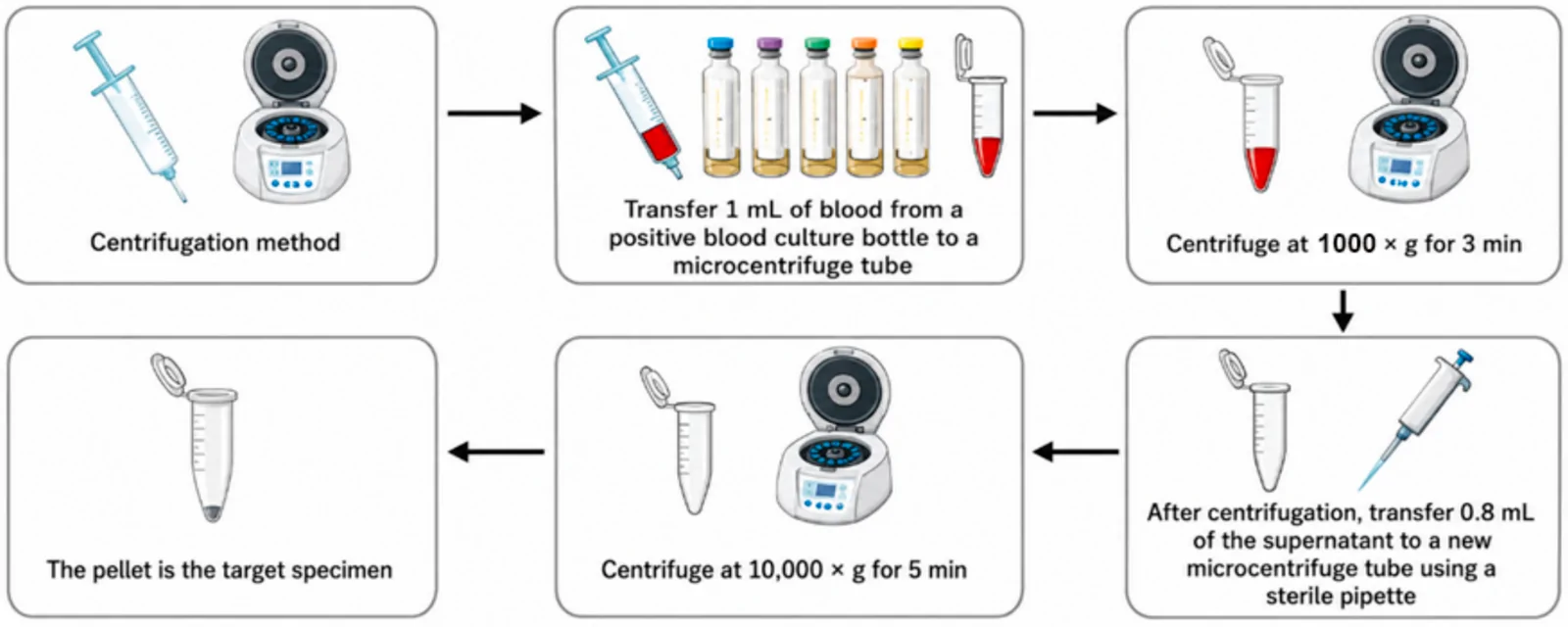
**Workflow of the direct centrifugation method for positive blood culture samples.** Schematic illustration of the direct centrifugation procedure used for sample preparation from positive blood culture bottles. Briefly, 1.0 mL of blood was aspirated from a positive blood culture bottle and transferred to a microcentrifuge tube. The sample was first centrifuged at 1000× *g* for 3 min to remove blood cells and debris. Subsequently, 0.8 mL of the supernatant was transferred to a new microcentrifuge tube and centrifuged at 10,000× *g* for 5 min. After removal of the supernatant, the resulting pellet was collected and used as the test specimen for downstream analysis.

A 1.0 mL aliquot was collected from each positive blood culture bottle and centrifuged at 1000× *g* for 3 min to remove blood cells and debris. Then, 0.8 mL of the supernatant was transferred to a new microcentrifuge tube and centrifuged at 10,000× *g* for 5 min. The resulting pellet was collected, adjusted to an appropriate bacterial suspension density, and directly subjected to antimicrobial susceptibility testing (AST) using the VITEK II system made by bioMérieux (Marcy-l’Étoile, France) with susceptibility cards selected according to the Gram stain results. Species identification was confirmed the following day by the standard workflow using VITEK MS (MALDI-TOF MS), and the identification results were subsequently entered into the VITEK II system for final interpretation (Figure 1).

### 2.4. Standard Routine Laboratory Workflow

**Figure 2:**
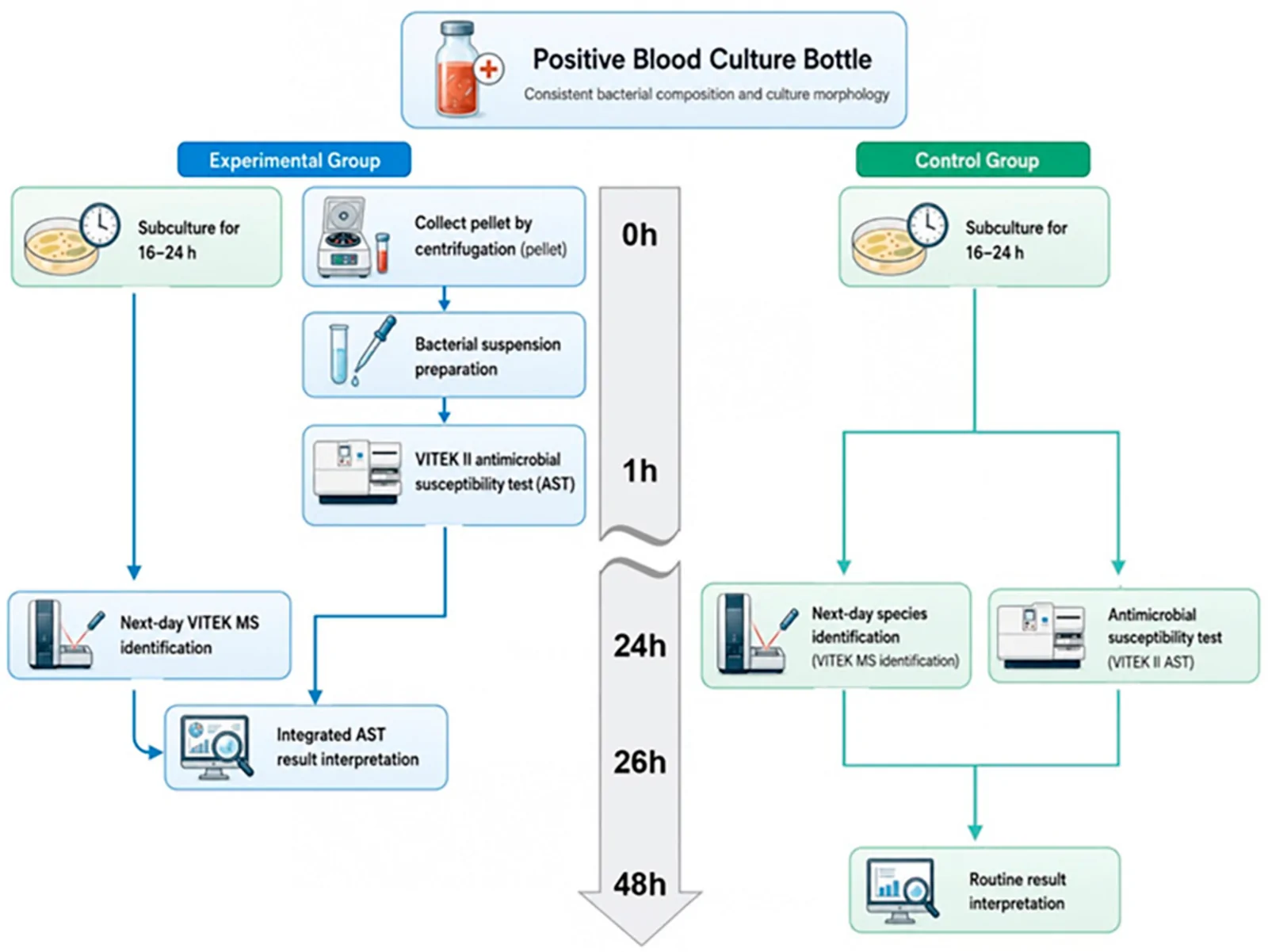
**Workflow comparison of direct centrifugation-based antimicrobial susceptibility testing and the conventional culture-based method for positive blood cultures.** Positive blood culture bottles with consistent bacterial composition and culture morphology were processed using either the experimental direct method or the routine control workflow. In the experimental group, aliquots from positive blood culture bottles were subcultured for next-day VITEK MS species identification, while bacterial pellets were simultaneously collected by centrifugation for bacterial suspension preparation and direct VITEK II antimicrobial susceptibility testing (AST). Integrated AST interpretation was subsequently performed by combining next-day species identification with direct AST results. In the control group, positive blood cultures were subcultured for 16–24 h before routine next-day VITEK MS identification and VITEK II AST, followed by standard result interpretation. The central timeline is schematic and not drawn to scale; the break between 1 h and 24 h indicates a non-proportional time interval.

The standard routine laboratory workflow served as the control method. Positive blood culture broths were subcultured onto solid agar media and incubated at 35 °C in 5% CO_2_ for 16–24 h. After incubation, a single colony was selected for species identification using VITEK MS (MALDI-TOF MS) made by bioMérieux (Marcy-l’Étoile, France). The same colony was then used to prepare the bacterial suspension for AST using the VITEK II system made by bioMérieux (Marcy-l’Étoile, France). The procedures are depicted in the right section of Figure 2.

### 2.5. Performance Criteria and Acceptance Thresholds

Performance metrics and acceptance criteria were established according to the Cumitech 31A guideline (Verification and Validation of Procedures in the Clinical Microbiology Laboratory) [18]. Essential agreement (EA) was defined as MIC results within one twofold dilution between the direct centrifugation and standard methods, whereas categorical agreement (CA) indicated concordant susceptible, intermediate, or resistant classifications. Both EA and CA were required to be ≥90%. A very major error (VME) was defined as a susceptible result obtained by the direct method when the standard method indicated resistance, whereas a major error (ME) was defined as a resistant result obtained by the direct method when the standard method indicated susceptibility. A minor error (mE) was defined as a discrepancy involving an intermediate result with the other method reporting either susceptibility or resistance. VME and ME rates were calculated using standard-method-resistant and standard-method-susceptible results as their respective denominators, while the mE rate was calculated using all tested organism–antimicrobial combinations. The acceptance thresholds were ≤3% for VME and ME rates and ≤10% for the mE rate.

### 2.6. Data Analysis and Evaluation

The agreement between the direct centrifugation method and the standard routine workflow in antimicrobial susceptibility testing results was assessed by determining categorical agreement (CA), essential agreement (EA), and rates of clinical interpretation errors. These analyses were performed to evaluate the clinical applicability of the shortened testing workflow for rapid diagnosis.

### 2.7. Turnaround Time Analysis and Statistical Methods

Turnaround time (TAT) was defined as the interval from sample acceptance by the laboratory to the reporting of the final antimicrobial susceptibility testing (AST) results. TAT was compared between the standard routine workflow and the direct centrifugation method. Continuous variables were summarized as mean ± standard deviation (SD), and the observed time intervals were additionally reported as ranges. TAT was compared between the standard workflow and the direct centrifugation method using a paired *t*-test because both workflows were applied to the same clinical specimens. Paired *t*-test analyses were also performed separately for Gram-negative bacteria (GNB) and Gram-positive cocci (GPC). A *p* value < 0.05 was considered statistically significant. All statistical analyses were performed using SPSS Statistics, version 21.0 (IBM Corp., Armonk, NY, USA).

## 3. Results

### 3.1. Study Population and Agreement of Antimicrobial Susceptibility Testing Results

Between January and September 2025, a total of 328 positive blood culture bottles showing a single microbial morphology on Gram staining were collected. Two samples were excluded because they did not meet the inclusion criteria: one was confirmed as a mixed culture after subculture, and the other yielded an insufficient bacterial pellet after centrifugation to prepare an appropriate suspension for antimicrobial susceptibility testing (AST). Consequently, 328 clinical isolates were included in the following analysis. AST results obtained by the direct centrifugation method were compared with those generated by the standard routine workflow for the 328 evaluable isolates. A total of 4393 antimicrobial–organism combinations were assessed.

### 3.2. Species-Specific Agreement Analysis for Gram-Negative Bacteria

Among the 168 Gram-negative bacterial isolates, the direct centrifugation method demonstrated high overall agreement with the standard routine workflow, with an essential agreement (EA) of 97.77% and a categorical agreement (CA) of 98.53% (Table 1). The overall very major error (VME), major error (ME), and minor error (mE) rates were 0.81%, 0.14%, and 0.60%, respectively, all within the Cumitech 31A acceptance criteria.

The two most frequently isolated species, *Escherichia coli* (n = 85) and *Klebsiella pneumoniae* (n = 40), demonstrated high concordance, with EA and CA values exceeding 98%. No VMEs were observed for *K. pneumoniae*. Most less frequently isolated species also showed acceptable agreement; however, *Acinetobacter lwoffii* (n = 5) had an EA of 88.57%, CA of 90.00%, and a VME rate of 4.35%, while *Salmonella* spp. (n = 4) had an EA and CA of 95.59% and a VME rate of 5.26%. Because these elevated VME rates were based on small numbers of isolates and discordant results, the corresponding species-specific estimates should be interpreted cautiously.

Antimicrobial-specific analysis comprised 2689 organism–antimicrobial tests and showed an overall categorical agreement of 99.1% (Supplementary Table S1). Four VMEs were identified, involving ceftazidime, colistin, piperacillin/tazobactam, and trimethoprim/sulfamethoxazole, with one VME for each agent. No VMEs were observed for ertapenem, imipenem, or meropenem. The lowest antimicrobial-specific agreement was observed for imipenem (96.3%), primarily because of six minor errors.

### 3.3. Species-Specific Agreement Analysis for Gram-Positive Cocci

A total of 160 Gram-positive cocci isolates were evaluated. The direct centrifugation method achieved an overall EA of 93.29% and CA of 95.91% compared with the standard routine workflow (Table 2). The overall VME, ME, and mE rates were 3.47%, 1.08%, and 0.94%, respectively. Antimicrobial-specific analysis comprised 1704 organism–antimicrobial tests and showed an overall categorical agreement of 97.1% (Supplementary Table S2). Although EA, CA, ME, and mE met the Cumitech 31A acceptance criteria, the overall VME rate slightly exceeded the predefined threshold of 3%.

At the species level, *Staphylococcus aureus* (n = 32) showed an EA of 92.56% and CA of 97.52%, whereas *Staphylococcus epidermidis* (n = 24) showed an EA of 92.40% and CA of 94.68%. Their VME rates were 4.71% and 6.03%, respectively. *Coagulase-negative staphylococci* (CNS, n = 62) demonstrated an EA of 91.48% and CA of 94.37%, with a VME rate of 1.79%. *Staphylococcus saprophyticus* (n = 8) showed an EA of 95.45%, CA of 97.73%, and a VME rate of 5.41%.

Among enterococci, *Enterococcus faecalis* (n = 9), *Enterococcus faecium* (n = 5), and *Enterococcus raffinosus* (n = 5) showed CA values ranging from 96.00% to 98.00%. No VMEs were observed for *E. faecium*, whereas the VME rates for *E. faecalis* and *E. raffinosus* were 5.88% and 2.78%, respectively. Streptococcal isolates generally showed high agreement: *Streptococcus dysgalactiae* and *group B streptococci* (GBS) both achieved 100% CA without observed errors. Other *Streptococcus* spp. (n = 9) achieved a CA of 96.61%, although the VME rate was 8.70%. Species-specific results based on small isolate numbers should therefore be interpreted cautiously.

Across 1704 Gram-positive organism–antimicrobial tests, the overall categorical agreement was 97.1%, with 23 VMEs, 11 MEs, and 16 minor errors (Supplementary Table S2). VMEs were most frequently observed with trimethoprim/sulfamethoxazole (n = 6) and clindamycin (n = 4), followed by penicillin (n = 3). Two VMEs each were identified for ciprofloxacin, gentamicin, levofloxacin, and oxacillin, whereas erythromycin and ampicillin each accounted for one VME. No VMEs were observed for vancomycin, linezolid, or teicoplanin. Gentamicin showed the lowest categorical agreement (87.9%), largely because of 14 minor errors.

### 3.4. Combined Analysis of AST Agreement in GNB and GPC Isolates

Among 168 Gram-negative bacterial and 160 Gram-positive coccal isolates, the direct centrifugation method demonstrated high overall agreement with the standard routine workflow. Of 4393 organism–antimicrobial comparisons, 4320 were categorically concordant, yielding an overall categorical agreement of 98.34% and an estimated essential agreement of 96.03%. A total of 27 very major errors (VMEs), 14 major errors (MEs), and 32 minor errors were identified. The combined ME rate was approximately 0.44% (14/3162 reference-susceptible results), with rates of 0.14% for Gram-negative bacteria and 1.08% for Gram-positive cocci.

Most VMEs occurred among Gram-positive cocci (23/27), resulting in an overall GPC VME rate of 3.47%, slightly exceeding the predefined 3% criterion. In contrast, only four VMEs occurred among Gram-negative bacteria. Following expansion from two to eight isolates, *Staphylococcus saprophyticus* showed an EA of 95.45%, CA of 97.73%, and no MEs. Overall, the direct centrifugation method showed strong concordance, particularly for Gram-negative bacteria; however, its performance was not uniformly acceptable across all error categories, and organism–antimicrobial combinations associated with VMEs require further validation.

### 3.5. Turnaround Time Comparison Between the Standard Workflow and the Direct Centrifugation Method

The direct centrifugation method significantly reduced the TAT for AST compared with the standard routine workflow (Table 3). TAT was defined as the interval from the time of specimen receipt in the laboratory to final AST result reporting. Among the 314 positive blood culture specimens analyzed in each workflow, the mean TAT decreased from 48.74 ± 2.83 h using the standard workflow to 25.13 ± 2.9 h with the direct centrifugation method, representing an average reduction of 23.61 h (*p* < 0.0001).

### 3.6. Significantly Reduced AST Turnaround Time by Direct Centrifugation in Both GNB and GPC Isolates

**Figure 3:**
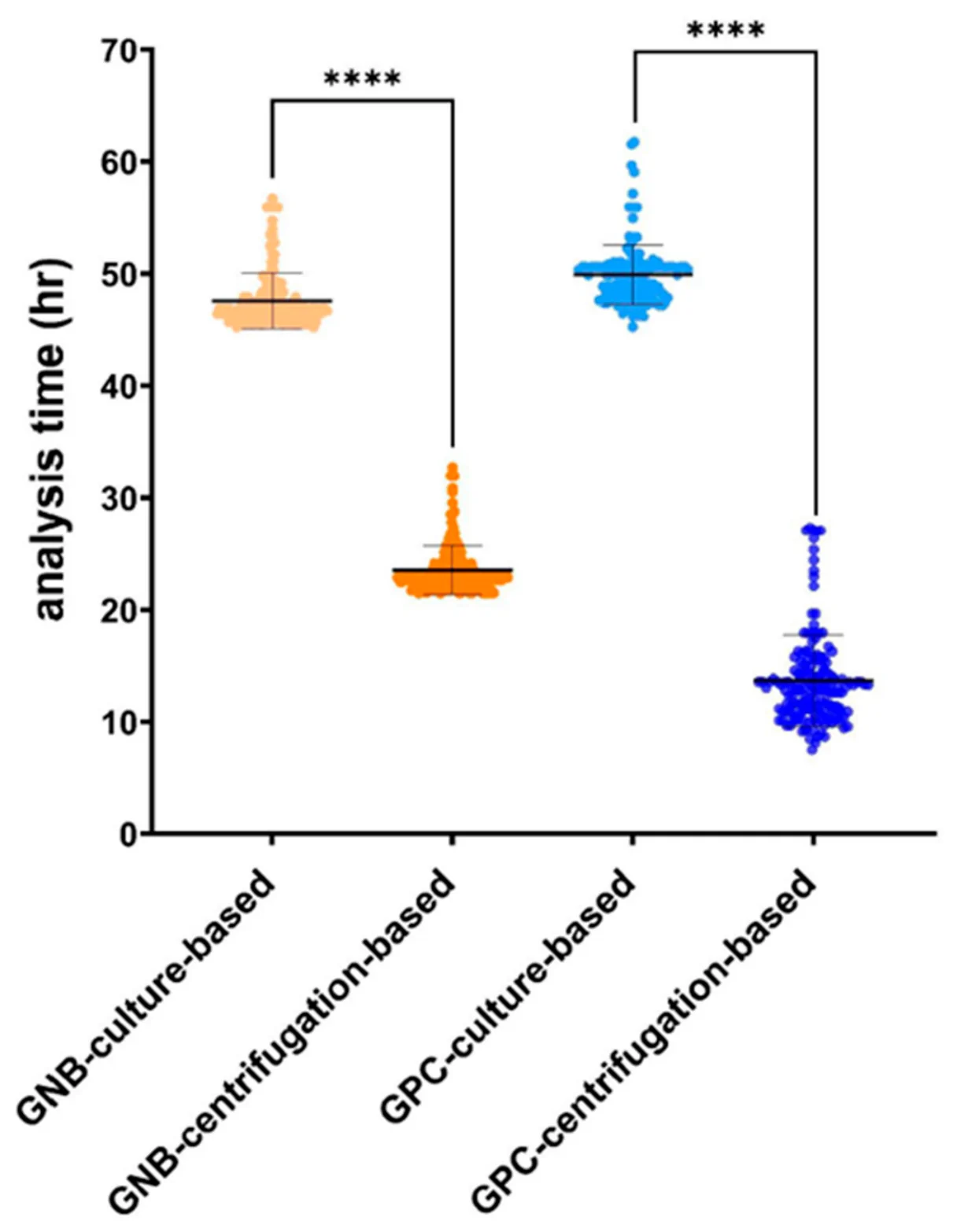
**Comparison of antimicrobial susceptibility testing turnaround time between the conventional culture-based workflow and the direct centrifugation-based workflow.** The turnaround time from specimen receipt in the laboratory to final AST result reporting was compared between the conventional culture-based method and the direct centrifugation-based method for Gram-negative bacilli (GNB) and Gram-positive cocci (GPC). Each dot represents an individual sample. Horizontal bars indicate the mean, and error bars represent the standard deviation. The direct centrifugation-based workflow significantly reduced the TAT compared with the conventional culture-based workflow in both GNB and GPC groups. Statistical significance was determined using a paired *t*-test. **** *p* < 0.0001.

To further evaluate whether the time-saving effect of the direct centrifugation-based workflow was consistent across different bacterial classifications, analysis time was compared separately between GNB and GPC isolates. As shown in Figure 3, the direct centrifugation-based workflow significantly shortened the analysis time compared with the conventional culture-based workflow in both GNB and GPC groups. For GNB isolates, the culture-based workflow required approximately 45 h for analysis, whereas the centrifugation-based method reduced the analysis time to approximately 23 h. A similar trend was observed for GPC isolates, in which the culture-based workflow generally required approximately 50 h, while the centrifugation-based method substantially reduced the analysis time to approximately 25 h.

The reduction in analysis time was statistically significant for both bacterial groups, with highly significant differences between culture-based and centrifugation-based workflows in GNB and GPC isolates with *p* values < 0.0001. These findings demonstrate that the direct centrifugation-based method markedly accelerates the workflow and provides a consistent reduction in reporting time across both GNB and GPC bloodstream isolates.

## 4. Discussion

The present study demonstrates that the direct centrifugation method provides high overall agreement with the standard workflow, particularly for Gram-negative organisms, where both essential agreement (EA ~98%) and categorical agreement (CA ~99%) exceeded established acceptance criteria. These findings are consistent with prior reports showing that direct antimicrobial susceptibility testing (AST) from positive blood cultures can achieve CA values in the range of 92–97% for both Gram-negative and Gram-positive bacteria when compared with conventional methods [19]. The particularly strong performance observed in common pathogens such as *Escherichia coli* and *Klebsiella pneumoniae* aligns with the notion that standardized inoculum preparation and robust growth characteristics in *Enterobacterales* facilitate reliable phenotypic AST. In contrast, the slightly reduced agreement in less frequently encountered species likely reflects both biological variability and statistical instability due to small sample sizes, a limitation also noted in previous evaluations of direct AST methodologies.

Although overall performance for Gram-positive cocci (GPC) remained within acceptable validation thresholds, the higher major error (ME) rates compared with Gram-negative organisms warrant attention. Similar trends have been reported in rapid AST platforms, where Gram-positive bacteria—particularly coagulase-negative staphylococci and less common species—show greater variability due to slower growth, heterogeneous resistance expression, and challenges in inoculum standardization [20]. Importantly, key pathogens such as *Staphylococcus aureus* and *enterococci* maintained high agreement and low error rates, supporting the clinical reliability of the method for major bloodstream infections. In contrast, reduced performance in rare species, such as *Staphylococcus saprophyticus* and *Enterococcus raffinosus*, highlights the need for cautious interpretation and, when necessary, confirmatory testing, consistent with prior reports emphasizing species-specific optimization in direct AST approaches [21].

To address this limitation, our study implemented an integrated workflow in which direct antimicrobial susceptibility testing was performed in parallel with conventional subculture-based identification. Specifically, while the direct centrifugation method enabled rapid AST initiation, isolates were simultaneously subcultured and subsequently identified using VITEK MS (MALDI-TOF MS) from single colonies after 16–24 h of incubation [22]. The confirmed organism identification was then retrospectively linked to the AST results generated by the VITEK II system [23]. This hybrid approach effectively compensates for the lower identification success associated with direct methods while preserving the advantage of accelerated susceptibility testing.

Such a combined strategy has been advocated in previous studies, which emphasize that direct methods should be integrated with, rather than replace, standard microbiological workflows to ensure diagnostic accuracy and clinical reliability [24]. By maintaining confirmatory identification through conventional culture, our approach ensures the robustness of final reports, particularly for clinically significant pathogens, while still achieving substantial reductions in turnaround time.

One of the most clinically significant findings of this study is the substantial reduction in TAT, with the direct centrifugation method shortening reporting time by approximately 23 h. This degree of improvement is consistent with previous studies reporting time savings of 18–24 h using direct AST workflows from positive blood cultures [19]. From a clinical perspective, earlier availability of AST results facilitates timely optimization of antimicrobial therapy, which is critical for improving outcomes in bloodstream infections and minimizing unnecessary broad-spectrum antibiotic use. It should be addressed that only a small number of isolates were included for certain species, such as *Acinetobacter lwoffii*, *Staphylococcus saprophyticus*, and *Enterococcus raffinosus*, and the mechanisms underlying discrepancies in these organisms require further investigation with larger sample sizes. Importantly, in the context of routine clinical implementation, potential workflow limitations can be effectively mitigated. Laboratories can readily revert to standard operating procedures using the original subcultured colonies, thereby maintaining consistency with conventional reporting timelines. This integrated strategy ensures that clinical decision-making remains uninterrupted while preserving both efficiency and diagnostic reliability.

This study did not formally assess costs or workload, including consumable use, hands-on time, processing delays, repeat testing, or Gram stain-guided card-selection errors. One mixed culture and one sample with an insufficient bacterial pellet required exclusion, indicating that conventional repeat testing may occasionally be necessary, reducing time savings and increasing costs. Because the observed TAT reduction may depend on staffing and laboratory operating hours, these implementation outcomes should be evaluated prospectively.

## 5. Conclusions

The direct centrifugation-based AST method demonstrated high agreement with the conventional workflow while substantially reducing the turnaround time for antimicrobial susceptibility results. The method showed particularly robust performance for Gram-negative bacteria, including clinically important bloodstream pathogens such as Escherichia coli and Klebsiella pneumoniae, while maintaining acceptable performance for Gram-positive cocci. By eliminating the need for additional subculture steps and enabling earlier susceptibility testing directly from positive blood cultures, this approach has the potential to provide clinically actionable AST results nearly one day earlier than conventional methods. Integration of this simple and practical workflow into routine clinical microbiology laboratories may facilitate earlier optimization of antimicrobial therapy, strengthen antimicrobial stewardship, and ultimately improve the management of patients with bloodstream infections and sepsis. Further multicenter studies incorporating a broader range of bacterial species and resistance phenotypes are warranted to confirm its generalizability and clinical impact.

## Figures and Tables

**Table 1:** Species-specific agreement of antimicrobial susceptibility testing results for Gram-negative bacteria (GNB) between the direct centrifugation method and the standard routine workflow.

Species (GNB)	Cumitech 31A Criteria EA ≥ 90, CA ≥ 90, VME ≤ 3, ME ≤ 3, mE ≤ 10					
	Amount	EA (%)	CA (%)	VME (%)	ME (%)	mE (%)
*Escherichia coli*	85	98.27	98.55	0.58	0.17	0.43
*Klebsiella pneumoniae*	40	98.53	99.41	0.00	0.00	0.15
*Klebsiella aerogenes*	4	95.59	98.53	0.00	0.00	1.47
*Serratia marcescens*	8	95.59	98.53	0.00	0.00	0.74
*Acinetobacter baumannii complex*	2	92.86	100	0.00	0.00	0.00
*Acinetobacter* spp.	1	100	100	0.00	0.00	0.00
*Acinetobacter lwoffii*	5	88.57	90.00	4.35	0.00	7.14
*Achromobacter* spp.	1	91.67	91.67	0.00	0.00	0.00
*Sphingomonas paucimobilis*	1	100	100	0.00	0.00	0.00
*Chryseobacterium indologenes*	1	100	100	0.00	0.00	0.00
*Citrobacter koseri*	2	100	100	0.00	0.00	0.00
*Enterobacter cloacae*	2	94.12	100	0.00	0.00	0.00
*Elizabethkingia meningoseptica*	1	100	100	0.00	0.00	0.00
*Proteus mirabilis*	4	98.44	98.44	0.00	0.00	1.59
*Providencia stuartii*	1	100	100	0.00	0.00	0.00
*Morganella morganii*	1	100	100	0.00	0.00	0.00
*Pseudomonas aeruginosa*	3	100	100	0.00	0.00	0.00
*Raoultella ornithinolytica*	1	100	100	0.00	0.00	0.00
*Salmonella* spp.	4	95.59	95.59	5.26	2.27	1.56
*Stenotrophomonas maltophilia*	1	100	100	0.00	0.00	0.00
Total	168	97.77	98.53	0.81	0.14	0.60

Abbreviations: essential agreement (EA), categorical agreement (CA), very major error (VME), major error (ME), and minor error (mE).

**Table 2:** Species-specific agreement of antimicrobial susceptibility testing results for Gram-positive cocci (GPC) between the direct centrifugation method and the standard routine workflow.

Species (GPC)	Cumitech 31A Criteria EA ≥ 90, CA ≥ 90, VME ≤ 3, ME ≤ 3, mE ≤ 10					
	Amount	EA (%)	CA (%)	VME (%)	ME (%)	mE (%)
*Staphylococcus aureus*	32	92.56	97.52	4.71	0.00	0.85
*Staphylococcus epidermidis*	24	92.40	94.68	6.03	2.82	0.76
*CNS*	62	91.48	94.37	1.79	2.10	1.61
*Staphylococcus saprophyticus*	8	95.45	97.73	5.41	0.00	0.00
*Enterococcus faecalis*	9	97.78	97.78	5.88	0.00	0.00
*Streptococcus dysgalactiae*	3	97.44	100	0.00	0.00	0.00
*Streptococcus* spp.	9	96.61	96.61	8.70	0.00	0.92
*GBS*	3	100	100	0.00	0.00	0.00
*Enterococcus faecium*	5	98.00	98.00	0.00	0.00	0.00
*Enterococcus raffinosus*	5	96.00	96.00	2.78	0.00	0.00
Total	160	93.29	95.91	3.47	1.08	0.94

Abbreviations: essential agreement (EA), categorical agreement (CA), very major error (VME), major error (ME), and minor error (mE). coagulase-negative Staphylococci (CNS), group B streptococci (GBS).

**Table 3:** Comparison of turnaround time for antimicrobial susceptibility testing results between the standard workflow and the direct centrifugation method.

Workflow	N	Time from Positive Blood Culture to AST Report (Hours, Range)	Mean ± SD (Hours)	*p* Value
Standard routine workflow	328	45.22–61.8	48.74 ± 2.83	
Direct centrifugation method	328	21.42–36.13	25.13 ± 2.9	<0.0001

TAT was defined as the interval from the time that specimen receipt in the laboratory to final AST result reporting. Data are presented as range and mean ± standard deviation (SD).

## Data Availability

The data presented in this study are available on request from the corresponding authors.

## Supplementary Materials

The following supporting information can be downloaded at: https://www.mdpi.com/article/10.3390/diagnostics16183007/s1, Table S1: Comparison of Minimum Inhibitory Concentration (MIC) Agreement and Error Counts Between the Direct Centrifugation and Standard Antimicrobial Susceptibility Testing Methods by Antimicrobial Agent for Gram-Negative Bacteria; Table S2: Comparison of Minimum Inhibitory Concentration (MIC) Agreement and Error Counts Between the Direct Centrifugation and Standard Antimicrobial Susceptibility Testing Methods by Antimicrobial Agent for Gram-Positive Bacteria.
