# Supplementary material for: Rapid Antimicrobial Susceptibility Testing Using Direct Centrifugation Significantly Reduces Turnaround Time While Maintaining High Diagnostic Accuracy in Bloodstream Infections
Source: Diagnostics (Basel). 2026 Sep 17;16(18):3007. doi: 10.3390/diagnostics16183007 (PMC13605901; doi:10.3390/diagnostics16183007)
Supplement: Supplementary file 1 [file diagnostics-16-03007-s001.zip › diagnostics-4518742-supplementary.pdf]

Supplementary Table S1. Comparison of Minimum Inhibitory Concentration (MIC) Agreement and Error Counts Between the Direct Centrifugation and Standard Antimicrobial Susceptibility Testing Methods by Antimicrobial Agent for Gram-Negative Bacteria.

GNB					
**Antibiotics**	Counts	Categorical agreement, n (%)	(VME)	(ME)	(mE)
**Amikacin**	167	167(100.0)	0	0	0
**Ampicillin/Sulbactam**	160	156(97.5)	0	1	3
**Cefazolin**	68	68(100.0)	0	0	0
**Cefepime**	167	165(98.8)	0	0	2
**Cefmetazole**	152	151(99.3)	0	0	1
**Cefotaxime**	166	166(100.0)	0	0	0
**Ceftazidime**	167	165(98.8)	1	0	1
**Ciprofloxacin**	163	163(100.0)	0	0	0
**Colistin**	163	161(98.8)	1	1	0
**Ertapenem**	152	151(99.3)	0	1	0
**Gentamicin**	167	166(99.4)	0	0	1
**Imipenem**	161	155(96.3)	0	0	6
**Levofloxacin**	168	167(99.4)	0	0	1
**Meropenem**	167	167(100.0)	0	0	0
**Piperacillin/Tazobactam**	166	165(99.4)	1	0	0
**Tigecycline**	167	166(99.4)	0	0	1
**Trimethoprim/Sulfamethoxazole**	168	167(99.4)	1	0	0
**Total**	2689	2666(99.1)	4	3	16

Supplementary Table S2. Comparison of Minimum Inhibitory Concentration (MIC) Agreement and Error Counts Between the Direct Centrifugation and Standard Antimicrobial Susceptibility Testing Methods by Antimicrobial Agent for Gram-Positive Bacteria.

GPC					
**Antibiotics**	Counts	Categorical agreement, n (%)	(VME)	(ME)	(mE)
**Penicillin**	160	157(98.1)	3	0	0
**Ciprofloxacin**	145	143(98.6)	2	0	0
**Clindamycin**	141	132(93.6)	4	5	0
**Erythromycin**	160	156(97.5)	1	2	1
**Gentamicin**	140	123(87.9)	2	1	14
**Levofloxacin**	160	158(98.8)	2	0	0
**Linezolid**	160	160(100.0)	0	0	0
**Oxacillin**	126	121(96.0)	2	3	0
**Trimethoprim/Sulfamethoxazole**	132	126(95.5)	6	0	0
**Teicoplanin**	10	10(100.0)	0	0	0
**Vancomycin**	160	160(100.0)	0	0	0
**Tetracycline**	97	97(100.0)	0	0	0
**Chloramphenicol**	11	11(100.0)	0	0	0
**Cefotaxime**	15	15(100.0)	0	0	0
**Ampicillin**	34	32(94.1)	1	0	1
**Ceftriaxon**	15	15(100.0)	0	0	0
**Gentamicin High Level (synergy)**	19	19(100.0)	0	0	0
**Streptomycin High Level (synergy)**	19	19(100.0)	0	0	0
**Total**	1704	1654(97.1)	23	11	16
